# Patients with diffuse idiopathic skeletal hyperostosis have an increased burden of thoracic aortic calcifications

**DOI:** 10.1093/rap/rkac060

**Published:** 2022-08-10

**Authors:** Netanja I Harlianto, Jan Westerink, Marjolein E Hol, Rianne Wittenberg, Wouter Foppen, Pieternella H van der Veen, Bram van Ginneken, Jorrit-Jan Verlaan, Pim A de Jong, Firdaus A A Mohamed Hoesein, UCC-SMART Study Group, F W Asselbergs, H M Nathoe, G J de Borst, M L Bots, M I Geerlings, M H Emmelot, P A de Jong, T Leiner, A T Lely, N P van der Kaaij, L J Kappelle, Y M Ruigrok, M C Verhaar, F L J Visseren, J Westerink

**Affiliations:** Department of Radiology; Department of Orthopedics; Department of Vascular Medicine, University Medical Center Utrecht and Utrecht University, Utrecht; Department of Radiology; Department of Radiology, Netherlands Cancer Institute, Amsterdam; Department of Radiology; Department of Radiology; Department of Medical Imaging, Radboud University Medical Center, Nijmegen, The Netherlands; Department of Orthopedics; Department of Radiology; Department of Radiology

**Keywords:** atherosclerosis, bone formation, calcification, diffuse idiopathic skeletal hyperostosis, thoracic aortic calcification

## Abstract

**Objectives.:**

DISH has been associated with increased coronary artery calcifications and incident ischaemic stroke. The formation of bone along the spine may share pathways with calcium deposition in the aorta. We hypothesized that patients with DISH have increased vascular calcifications. Therefore we aimed to investigate the presence and extent of DISH in relation to thoracic aortic calcification (TAC) severity.

**Methods.:**

This cross-sectional study included 4703 patients from the Second Manifestation of ARTerial disease cohort, consisting of patients with cardiovascular events or risk factors for cardiovascular disease. Chest radiographs were scored for DISH using the Resnick criteria. Different severities of TAC were scored arbitrarily from no TAC to mild, moderate or severe TAC. Using multivariate logistic regression, the associations between DISH and TAC were analysed with adjustments for age, sex, BMI, diabetes, smoking status, non-high-density lipoprotein cholesterol, cholesterol lowering drug usage, renal function and blood pressure.

**Results.:**

A total of 442 patients (9.4%) had evidence of DISH and 1789 (38%) patients had TAC. The prevalence of DISH increased from 6.6% in the no TAC group to 10.8% in the mild, 14.3% in the moderate and 17.1% in the severe TAC group. After adjustments, DISH was significantly associated with the presence of TAC [odds ratio (OR) 1.46 [95% CI 1.17, 1.82)]. In multinomial analyses, DISH was associated with moderate TAC [OR 1.43 (95% CI 1.06, 1.93)] and severe TAC [OR 1.67 (95% CI 1.19, 2.36)].

**Conclusions.:**

Subjects with DISH have increased TACs, providing further evidence that patients with DISH have an increased burden of vascular calcifications.

Key messages

- The prevalence of DISH increases with thoracic aortic calcification (TAC) severity.
- Subjects with DISH have more TAC, which may elucidate the relationship between DISH and ischaemic stroke.
- Bone formation in DISH and vessel calcification may share aetiological pathways.

## Introduction

In 1950, Forestier and Rotés-Querol were the first to describe a case of enthesopathy and hyperostosis at the anterolateral part of the spine, which later came to be known as DISH [1]. The most common manifestation of DISH is the formation of new bone in the spinal column, which can also be observed to a lesser extent in the peripheral skeleton [2]. DISH is most frequently reported in patients >50 years of age, becomes more prevalent with increasing age and males are predominantly affected [2]. The exact developmental mechanism for DISH remains undetermined, but a strong metabolic component with low-grade inflammation is likely involved, as DISH is associated with diabetes, obesity and metabolic syndrome [2, 3]. DISH may compress structures near the spine, resulting in myelopathy and radiculopathy [4]. Interestingly, DISH has been identified to be an independent predictor for ischaemic stroke [5].

Thoracic aortic calcifications (TACs) are common and mostly regarded as incidental observations on chest radiographs and CT scans [6]. Chest radiographs are more easily performed and accessible, whereas CT scans are able to provide a numerical estimate of calcification. In the thoracic aorta, TACs are associated with thickening of the arterial wall and stiffening of the aorta [7]. The stiffening leads to a dysfunctional Windkessel effect [7], in which chronic damage in the peripheral legs, kidneys and brain may occur. Indeed, TACs have been reported to be an independent predictor for cardiovascular events and TACs have been associated with unfavourable mortality outcomes in large epidemiological studies [8, 9]. The deposition of calcium in the arterial walls shows many histological similarities to that of bone formation [10]. Research investigating the relation between DISH and vascular calcification, however, is limited.

Oudkerk *et al.* [11] previously studied DISH and the burden of coronary artery calcification in smokers using the Agatston method. DISH was significantly associated with more coronary calcifications, which remained significant after extensive confounder correction. Another study derived from the general population found an association between the presence of DISH and abdominal aortic calcifications on radiographs. However, this relation became attenuated after correcting for age [12]. Additional studies exploring these relations may provide more insights into the potential overlap between bone formation in DISH and calcifications in blood vessels. Furthermore, a relation between these processes may provide more insights into DISH as a risk factor for the development of cardiovascular disease, including ischaemic stroke. The association between DISH and TAC is still unknown, and no previous study has assessed the severity of DISH in relation to the presence of calcification.

As these two processes share common aetiological pathways, we hypothesize that subjects with DISH have more TACs. Therefore the objective of the current study was to investigate the relation between the presence and severity of DISH and the presence and severity of TAC.

## Materials and methods

### Study population

This study was conducted in accordance with the Strengthening the Reporting of Observational Studies in Epidemiology guidelines [13]. Our study population is derived from the Second Manifestations of ARTerial disease (UCC-SMART) study, an ongoing prospective cohort study that started in 1996, following patients between the ages of 18 and 79 years with either manifest or risk factors for vascular disease. The UCC-SMART study was conducted in accordance with the Declaration of Helsinki and was approved by the local medical ethics committee (NL45885.041.13) and all included patients provided written informed consent. Patients with a digital chest radiograph within 3 months of inclusion in the UCC-SMART study were identified. Subsequently we excluded patients in which DISH and/or TAC could not be adequately assessed.

### Physical and laboratory measurements

Extensive vascular screening was performed for all included patients in the UCC-SMART study: patients were asked to complete a health questionnaire covering medical history, risk factors, smoking and drinking habits and prescribed drugs. A standardized diagnostic protocol was followed in the UCC-SMART study comprising physical examination and laboratory testing in a fasting state [14]. BMI was calculated as weight divided by height squared (kg/m^2^). Blood pressure (BP) was measured using a non-random sphygmomanometer and was performed three times at the right and left upper arm in an upright position with an interval of 30 s. The mean of the last two measurements from the highest arm was used. Hypertension was defined as systolic blood pressure (SBP) ≥140 mmHg and/or diastolic blood pressure (DBP) ≥90 mmHg and/or use of antihypertensive medication. Pulse pressure was defined as the difference between the brachial SBP and DBP. Fasting blood samples were available for measurements of blood lipids, haemoglobin A1c (HbA1c), glucose, high-sensitivity C-reactive protein (hs-CRP) and creatinine levels. Hyperlipidaemia was defined as low-density lipoprotein (LDL) cholesterol ≥2.6 mmol/l [15]. Renal function was estimated using the Chronic Kidney Disease Epidemiology Collaboration equation [16]. Diabetes mellitus at baseline was defined as either a referral diagnosis of diabetes, self-reported diabetes including the use of glucose-lowering agents, glucose ≥11.1 mmol/l or initiation of glucose-lowering treatment within 1 year after inclusion with glucose ≥7.0 mmol/l at baseline. Metabolic syndrome was defined according to the National Cholesterol Education Program criteria [17].

### Assessment of DISH and TAC

Chest radiographs were assessed for the presence of DISH by a group of six readers from the Department of Radiology of our institution, all certified to independently read chest radiographs (Entrustable Professional Activity level 4 or 5 for chest radiograph interpretation). DISH was diagnosed using the criteria from Resnick and Niwayama [18] following the presence of flowing bridging ossification of at least four contiguous vertebrae, (relative) preservation of the intervertebral disc height and the absence of apophyseal joint bony ankylosis. The severity of DISH was scored as described previously [19]: grade 1 DISH indicated flowing bridging osteophytes of four adjacent vertebral bodies, grade 2 DISH indicated flowing bridging osteophytes of five or six vertebral bodies and grade 3 DISH indicated flowing bridging osteophytes of seven or more vertebral bodies.

**Fig. 1:**
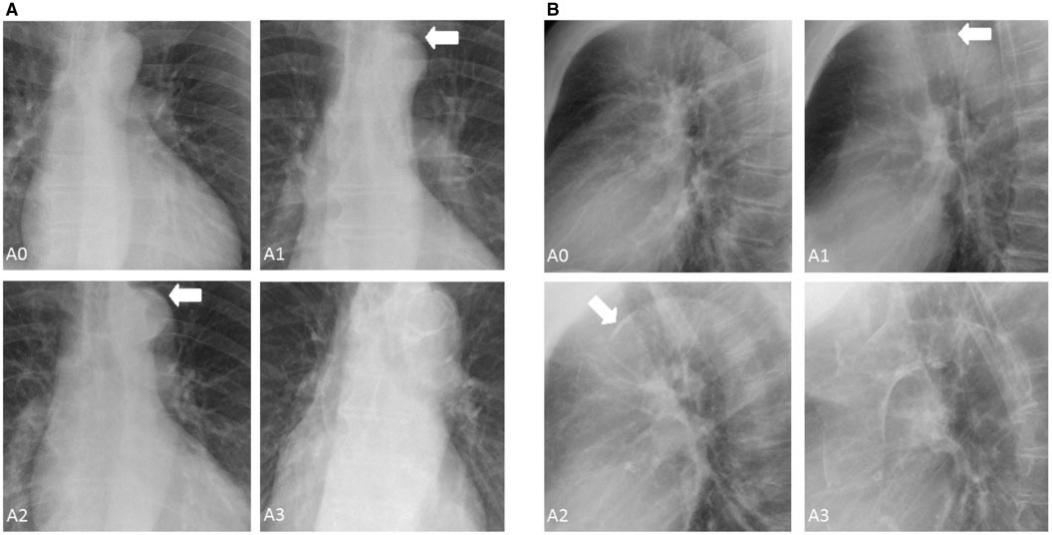
(A) Anteroposterior and (B) lateral radiographs illustrating different severities of TAC

The presence and severity of TAC were also scored on the chest radiographs using an arbitrary scale. TAC was classified into four categories: A0 (no TAC): no visible calcifications; A1 (mild TAC): borderline calcifications or mild calcification suspected; A2 (moderate TAC): clear calcification, multiple dots or one large calcification; A3 (severe TAC): extensive calcification (Figure 1A and B).

### Statistics

Normal distributed data were expressed using the mean and s.d. and categorical variables using frequency and percentage. Using logarithmic transformation, we transformed right-skewed data. Differences between groups were analysed using the Student’s *t*-test for normally distributed data and the chi-squared test for categorical data. The prevalence of DISH was compared between the different severities of TAC. Univariate logistic regression was performed with TAC (present/absent) as the outcome and the presence of DISH as an independent factor, stratified for the total DISH group and each severity of DISH. Using a stepwise-adjusted approach including confounder selection based on the literature and aetiologic considerations, we then performed multivariate logistic regression with adjustments for age and sex, and subsequently adjusted for BMI, renal function, BP, diabetes, smoking status, non-high-density lipoprotein (HDL) cholesterol and cholesterol-lowering drug use. Multinomial logistic regression was performed for the different severities of TAC as the outcome, with the total DISH group (present/absent) as an independent factor. This model was also adjusted for age and sex and subsequently for BMI, renal function, BP, diabetes, smoking status, non-HDL cholesterol and cholesterol-lowering drug use. All models were stated as odds ratios (ORs) with 95% CIs. In the sensitivity analysis, we performed additional analyses to evaluate the interaction effects between DISH and age and sex. We also evaluated the effect of a history of vascular disease on the relation between DISH and TAC using interaction analyses in regression modelling. Missing covariate data, including BMI (0.1%), non-HDL cholesterol (0.3%), SBP (0.1%) and renal function (0.3%) were imputed with single-regression imputation using the mice package. Statistical significance was set at *P* < 0.05. Data analysis was performed with R version 3.6.3 (R Foundation for Statistical Computing, Vienna, Austria).

## Results

### Baseline characteristics

A total of 4791 patients were identified, of which 88 were excluded due to technical image deficiencies (*n* = 44), only anterioposterior radiograph being available (*n* = 34) or poor image quality (*n* = 10), resulting in 4703 available patients for inclusion in the current study (Supplementary Fig. S1, available at *Rheumatology Advances in Practice* online). The mean age of our cohort was 58.4 years (s.d. 11.2), of which 69.7% was male. In our cohort, 442 (9.4%) patients had evidence of DISH, comprising 165 patients classified as grade 1, 143 patients as grade 2 and 134 patients as grade 3. The demographics of our study population between subjects with and without DISH are listed in Table 1. DISH patients were older (65.7 *vs* 57.6 years), more frequently male (85.7% *vs* 68%) and had a significantly higher BMI (28.6 *vs* 26.9 kg/m^2^), BP (146.3 *vs* 140.7 mmHg) and pulse pressure (63.4 *vs* 57.2 mmHg) compared with patients without DISH. Furthermore, patients with DISH were observed to have more type 2 diabetes (31% *vs* 20.8%), hypertension (31% *vs* 24.1%) and vascular disease (75.3% *vs* 67.4%).

**Table 1:** Baseline patient characteristics

Variable	Total group (*N* = 4703)	DISH (*n* = 442)	No DISH (*n* = 4261)	*P*-value
Age, years, mean (s.d.)	58.4 (11.2)	65.7 (7.8)	57.6 (11.2)	<0.001
Sex (male), %	69.7	85.7	68	<0.001
Any TAC, %	38	56.3	36.1	<0.001
Type 2 diabetes, %	21.7	31	20.8	<0.001
BMI, kg/m^2^, mean (s.d.)	27.1 (4.5)	28.6 (4.5)	26.9 (4.5)	<0.001
Glucose, mmol/l, mean (s.d.)	6.4 (1.9)	6.7 (1.6)	6.3 (1.9)	<0.001
HbA1c, %, mean (s.d.)	6 (1.1)	6.1 (1.0)	5.9 (1.1)	0.009
eGFR, mL/min/1.73 m^2^, mean (s.d.)	78.4 (19.2)	73.2 (17.6)	79 (19.2)	<0.001
SBP, mmHg, mean (s.d.)	141.3 (21.7)	146.3 (22.4)	140.7 (21.6)	<0.001
Hypertension, %^a^	24.8	31	24.1	0.002
Pulse pressure, mmHg, mean (s.d.)	57.8 (15.3)	63.4 (16)	57.2 (15.2)	<0.001
Non-HDL cholesterol, mmol/l, mean (s.d.)	3.66 (1.30)	3.64 (1.57)	3.7 (1.27)	0.78
Hs-CRP, mg/l, mean (s.d.)^b^	1.08 (0.78)	1.16 (0.77)	1.06 (0.78)	0.30
Metabolic syndrome, %^a^	53.9	65.4	52.7	<0.001
Smoking (ever *vs* never), %^a^	73.1	76.9	72.7	0.048
Pack-years, mean (s.d.)	17.5 (19.5)	18.6 (19.9)	17.4 (19.4)	0.23
Alcohol usage (current *vs* former), %^a^	80.4	85.7	79.9	0.004
History of vascular disease, %^a^	68.1	75.3	67.4	0.002

a Percentages were calculated after excluding missing cases from the denominator.

b Log transformed.

### The presence of TACs in relation to DISH

A total of 1789 subjects had TAC, comprising 727 subjects with mild, 652 with moderate and 410 patients with severe TAC. Subjects with DISH more often had TAC compared with subjects without DISH (56.3% *vs* 36.1%; *P* < 0.001). Results of logistic regression analysis with the presence of any TAC as the outcome are listed in Table 2. DISH was positively associated with any TAC in the univariate analysis [OR 2.28 (95% CI 1.87, 2.78), *P* < 0.001], which remained statistically significant after adjustments were made for age, sex [OR 1.46 (95% CI 1.18, 1.81), *P* < 0.001] and cardiovascular risk factors [OR 1.46 (95% CI 1.17, 1.82), *P* < 0.001]. After stratifying by the severity of DISH, all grades of DISH were significantly associated with any TAC in the crude analysis. However, the severity of DISH did not display a clear association with the presence of any TAC, as these relations became attenuated and insignificant for grade 1 and 3 DISH after correcting for age and sex and cardiovascular risk factors. The prevalence of DISH increased with the severity of TAC when comparing the different groups: 10.8% of patients with mild TAC had evidence of DISH, which was 14.3% and 17.1% for the moderate and the most severe TAC group, respectively (Table 3).

**Table 2:** Risk factor analysis with presence of any TAC as the outcome

Variable	Units	Univariate model		Age and sex adjusted		Age, sex and cardiovascular risk factor adjusted	
		OR (95% CI)	*P*-value	OR (95% CI)	*P*-value	OR (95% CI)	*P*-value
Total DISH group	Present *vs* absent	2.28 (1.87, 2.78)	<0.001	1.46 (1.18, 1.81)	<0.001	1.46 (1.17, 1.82)^d^	<0.001
Grade 1 DISH	Present *vs* absent	1.92 (1.41, 2.63)	<0.001	1.30 (0.93, 1.82)	0.12	1.19 (0.84, 1.70)^d^	0.33
Grade 2 DISH	Present *vs* absent	2.91 (2.17, 2.63)	<0.001	2.00 (1.39, 2.89)	<0.001	2.05 (1.41, 3.00)^d^	<0.001
Grade 3 DISH	Present *vs* absent	2.18 (1.54, 3.09)	<0.001	1.21 (0.84, 1.74)	0.32	1.21 (0.82, 1.78)^d^	0.33
Age^a^	+1 year	1.09 (1.08, 1.10)	<0.001	1.09 (1.08, 1.10)	<0.001	1.1 (1.08, 1.11)	<0.001
Sex^b^	Male *vs* female	1.46 (1.28, 1.65)	<0.001	1.67 (1.45, 1.92)	<0.001	1.77 (1.53, 2.05)	<0.001
Type 2 diabetes mellitus	Present *vs* absent	1.32 (1.14, 1.52)	<0.001	1.15 (0.99, 1.34)	0.07	1.21 (0.67, 2.28)	0.55
BMI	+1 kg	0.98 (0.97, 0.99)	<0.001	0.96 (0.95, 0.98)	<0.001	0.97 (0.96, 0.99)	<0.001
Glucose	+1 mmol/l	1.05 (1.02, 1.08)	0.002	1.04 (1.01, 1.08)	0.02	1.03 (0.99, 1.08)	0.16
HbA1c	+1%	1.16 (1.09, 1.24)	<0.001	1.15 (1.07, 1.24)	<0.001	1.07 (0.97, 1.19)	0.16
Renal function	+1 ml/min/1.73 m^2^	0.98 (0.97, 0.98)	<0.001	1.00 (0.99, 1.01)	0.12	1.00 (0.99, 1.01)	0.09
SBP	+1 mmHg	1.01 (1.01, 1.01)	<0.001	1.00 (1.00, 1.01)	0.005	1.00 (1.00, 1.01)	0.01
Hypertension	Present *vs* absent	1.15 (1.00, 1.31)	0.049	1.06 (0.92, 1.24)	0.41	0.84 (0.68, 1.05)	0.14
Pulse pressure	+1 mmHg	1.03 (1.02, 1.03)	<0.001	1.01 (1.00, 1.02)	<0.001	1.02 (1.01, 1.03)	<0.001
Non HDL cholesterol	+1 mmol/l	1.00 (0.95, 1.04)	0.84	1.12 (1.07, 1.18)	<0.001	1.12 (1.06, 1.18)	<0.001
hs-CRP^c^	+1 log(mg/l)	1.14 (1.08, 1.21)	<0.001	1.10 (1.04, 1.17)	0.002	1.11 (1.01, 1.21)	0.03
Metabolic syndrome	Present *vs* absent	1.11 (0.99, 1.26)	0.38	1.09 (0.95, 1.24)	0.21	1.15 (0.98, 1.34)	0.08
Smoking	Current *vs* former	1.45 (1.27, 1.67)	<0.001	1.61 (1.38, 1.88)	<0.001	1.61 (1.38, 1.89)	<0.001
Pack-years	+1 pack-year	1.01 (1.00, 1.02)	<0.001	1.01 (1.00, 1.02)	<0.001	1.01 (1.00, 1.01)	<0.001
Alcohol use	Current *vs* former drinker	0.93 (0.80, 1.08)	0.35	1.06 (0.90, 1.27)	0.48	0.95 (0.79, 1.13)	0.55
History of vascular disease	Yes *vs* no	2.10 (1.82, 2.42)	<0.001	1.36 (1.15, 1.60)	<0.001	1.63 (1.37, 1.95)	<0.001

a Sex adjusted.

b Age adjusted.

c Log transformed.

d Additionally adjusted for lipid-lowering drug use.

Third model adjusted for age, sex, BMI, diabetes mellitus, smoking status, non-HDL cholesterol, renal function and BP.

**Table 3:** The prevalence of DISH in the different severity categories of TAC

	No TAC, *n* (%)	Mild TAC, *n* (%)	Moderate TAC, *n* (%)	Severe TAC, *n* (%)
DISH	193 (6.6)	86 (10.8)	93 (14.3)	70 (17.1)
No DISH	2721 (93.4)	641 (89.2)	559 (85.7)	340 (82.9)

The results of multinomial logistic regression with different severities of TAC as the outcome using subjects without TAC as reference category are listed in Table 4. DISH was significantly associated with all severities of TAC in the crude analysis. A clear increase in odds was observed with increasing categories of TAC from mild to severe. After adjusting for atherosclerotic risk factors and statin use, DISH was significantly associated with moderate TAC [OR 1.43 (95% CI 1.06, 1.93)] and severe TAC [OR 1.67 (95% CI 1.19, 2.36)]. These results remained unchanged after including a history of vascular disease in the third model (Supplementary Table S1, available at *Rheumatology Advances in Practice* online). In sensitivity analyses, no effect modification was observed between DISH and age (*P* for interaction = 0.08), sex (*P* for interaction = 0.59) or a history of vascular disease (*P* for interaction = 0.17).

**Table 4:** Multinomial logistic regression analysis with different TAC categories as the outcome

Variable	Mild TAC		Moderate TAC		Severe TAC	
	OR (95% CI)	*P*-value	OR (95% CI)	*P*-value	OR (95% CI)	*P*-value
DISH crude	1.89 (1.45, 2.47)	<0.001	2.35 (1.80, 3.05)	<0.001	2.90 (2.16, 3.90)	<0.001
DISH age and sex adjusted	1.32 (1.00, 1.74)	0.052	1.47 (1.11, 1.94)	0.007	1.76 (1.27, 2.43)	<0.001
DISH age, sex and cardiovascular risk factors adjusted	1.33 (0.99, 1.77)	0.055	1.43 (1.06, 1.93)	0.02	1.67 (1.19, 2.36)	0.003

Third model adjusted for age, sex, BMI, diabetes mellitus, smoking status, non-HDL cholesterol, renal function, BP and lipid-lowering drug use.

## Discussion

We aimed to study the relation between DISH and different severities of TAC in patients with increased risk for cardiovascular disease. We found that the presence of DISH was associated with the presence of TAC, which was independent of age, sex and atherosclerotic risk factors. These relations became stronger as the severity of TAC increased, which was also unaffected by age and sex in exploratory sensitivity analyses.

Overall, the prevalence of DISH in our cohort was 9.4%. When stratified by the extent of TAC, we observed an increasing prevalence of DISH, but the extent of ossification in DISH was not related to the extent of TAC.

The results of our study confirm the findings of previous work that DISH is associated with increased calcifications in blood vessels [11]. It is postulated that patients with DISH may be prone to form calcifications [5], which may not be limited to the arteries, as increased calcifications of the aortic valve have also been reported in DISH patients [20]. In addition, in a patient population undergoing total hip arthroplasty, patients classified with DISH were three times more likely to form ectopic bone around the hip arthroplasty following surgery, which significantly affected pain levels and caused more movement restriction of the hip joint, compared with subjects with less or no ossification [21]. In histological studies into the bony bridges of DISH, processes of both heterotopic ossification and dystrophic calcification have been described [22]. In our results, the magnitudes of ORs were similar for DISH and TAC between the age- and sex-adjusted model and the model adjusted for atherosclerotic risk factors. This may suggest that DISH and TAC are in fact the same processes at different stages in time.

Underlying genetic disorders may also cause heterotopic ossification and calcification at the paravertebral spine with a presentation similar to DISH. In some of these described patients, concomitant calcifications were observed in the brain, eyes or kidneys [23]. Whether patients with DISH have increased calcifications in these locations remains to be determined. Conversely, in disorders characterized by the formation of extensive calcification, such as pseudoxantoma elasticum or Fahr’s disease, it is still unknown whether these patients develop DISH over time. We do acknowledge that our study is cross-sectional in its design and that studies with longitudinal imaging data and preferably even experimental studies are needed to confirm our findings. All published studies evaluating calcification in DISH are cross-sectional in design. A recent study by Lantsman *et al.* [24] found no independent relation between DISH and coronary artery calcium scores. Furthermore, within our study cohort we did not find relations between DISH and incident myocardial infarction [5].

In our patient population, we previously showed that DISH is an independent predictor for ischaemic stroke, with DISH subjects having a 55% increased rate for ischaemic stroke independent of age, sex and cardiovascular risk factors [5]. Although largely speculation, the main findings of the current study may help in further elucidating the relation between DISH and ischaemic stroke. TAC has been identified as an independent predictor for ischaemic stroke in patient samples with increased risk and samples from the general population [25, 26].

Although the pathophysiology of DISH remains poorly understood, one possible pathway for bone formation in DISH is that of hyperinsulinemia, which may induce chondrogenesis and ossification near the spinal ligaments [2]. As DISH has been strongly associated with adipose tissue, another pathway may imply the involvement of low-grade inflammation in the pathological process of bone formation in DISH [18]. Likewise, calcification in blood vessels in the development of atherosclerosis is facilitated by increased processes of inflammation [27]. Also, there may be unknown genetic factors that make people prone to bone formation.

The nature of TAC is not well known, although older studies in human tissue suggest that these calcifications are often located in the tunica media [6]. It is evident that in medial arterial calcification (MAC), bone formation is seen in the latest stages of MAC, usually involving calcifications >3 mm or calcifications spanning the entire circumference of the blood vessel [28]. As such, TAC can influence the distensibility and pulse pressure (both determinants of arterial stiffness) and hence the normal Windkessel function of the aorta. Indeed, this is supported in our study, as DISH patients had higher SBP and pulse pressure compared with subjects without DISH, and both increased BP and pulse pressure were associated with the presence of TAC after adjustments. Conversely, some authors have shown bone-like calcification in peripheral atherosclerotic lesions, with the involvement of cells similar to osteoblasts and osteoclasts [29]. DISH and atherosclerosis may share processes of angiogenesis, given the overlap between the two disorders in various metabolic abnormalities, which supports the notion that DISH is more likely a syndrome than a disease [30].

Currently no treatment exits that is able to slow down or halt the progression of calcification in both DISH and in blood vessels. At this time, various interventions are being explored as potential treatments for arterial calcification, including bisphosphonates and myo-inositol hexaphosphate [31, 32]. We believe that it is important to gain a better aetiological understanding of bone-forming disorders in relation to cardiovascular disease. With the current study, we provide additional evidence to support further research into a possible shared aetiology between these two processes.

### Strengths and limitations

Strengths of our study include the uniform prospective data collection of our relatively large cohort, with accurate and systematic measurements of extensive cardiovascular confounding factors. Furthermore, our study is the first to evaluate the severity of DISH in relation to vascular calcification. The limitations of our study should also be noted. The Resnick criteria for DISH are arbitrary and some milder forms or earlier stages of DISH will be misclassified, as our study did not include early forms of DISH [33]. Second, as the design of our study is cross-sectional, caution should be exercised in drawing causal conclusions. Finally, although all readers were certified to read chest radiographs independently (four senior radiology residents and two cardiothoracic radiologists), we did not have the data available on observer variation.

## Conclusion

The presence of DISH is associated with the presence and severity of TACs. Our study provides further evidence that patients with DISH have more systemic excessive bone formation that can put these patients at an increased risk for cardiovascular disease.

## Supplementary Material

rkac060_Supplementary_Data

## Data Availability

The informed consent that was signed by the study participants is not compliant with publishing individual data in an open access institutional repository or as supporting information files with the published paper. However, a data request can be sent to the SMART Steering Committee at uccdatarequest@umcutrecht.nl.
