# Supplementary material for: Patients with diffuse idiopathic skeletal hyperostosis have an increased burden of thoracic aortic calcifications
Source: Rheumatol Adv Pract. 2022 Aug 10;6(2):rkac060. doi: 10.1093/rap/rkac060 (PMC9382268; doi:10.1093/rap/rkac060)
Supplement: rkac060_Supplementary_Data [file rkac060_supplementary_data.docx]

**Supplementary Material:**

Patients with diffuse idiopathic skeletal hyperostosis have an increased burden of thoracic aortic calcifications by Harlianto et al.


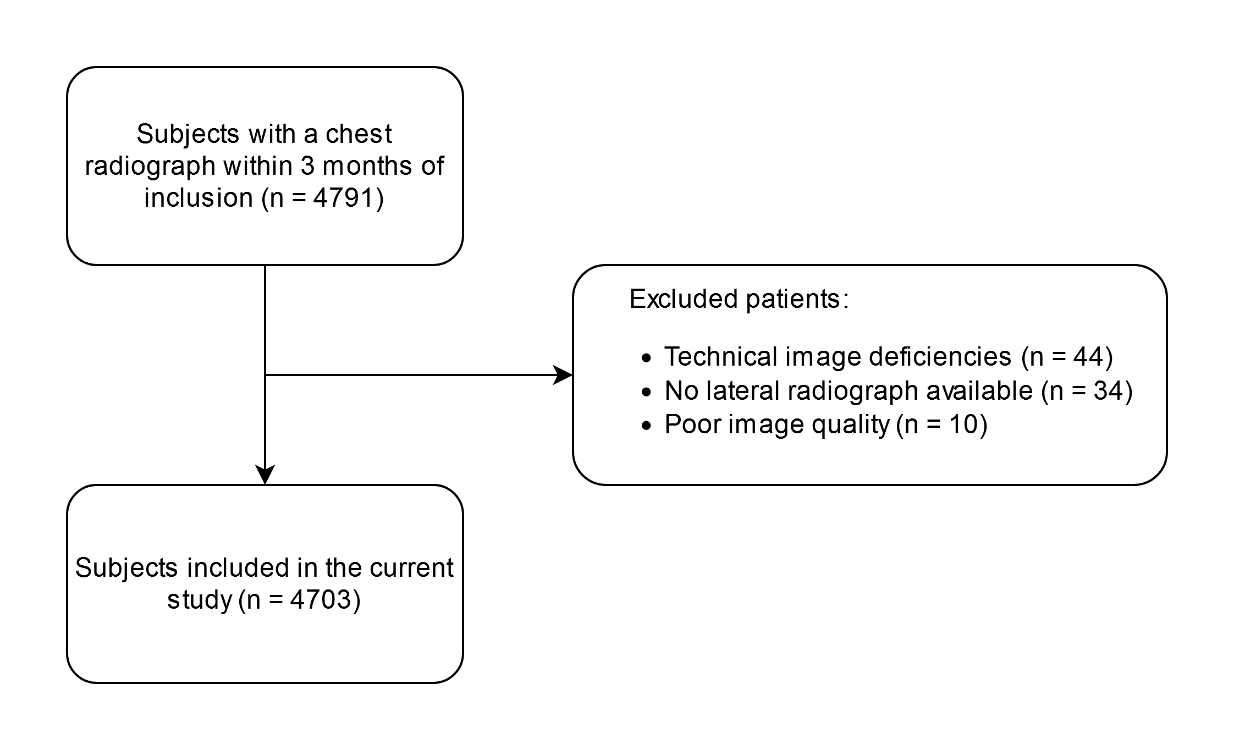


Supplementary Figure S1. Flowchart of patient selection

Supplementary Table S1. Sensitivity analyses with inclusion of a history of vascular disease in the final model.

|  | **Mild TAC** | | **Moderate TAC** | | **Severe TAC** | |
| --- | --- | --- | --- | --- | --- | --- |
| Variable | **OR (95%CI)** | **p-value** | **OR (95%CI)** | **p-value** | **OR (95%CI)** | **p-value** |
| Model 1 | 1.29 (0.97 - 1.71) | 0.08 | 1.54 (1.15 - 2.05) | 0.004 | 1.82 (1.30 - 2.54) | <0.001 |
| Model 2 | 1.31 (0.98 - 1.75) | 0.06 | 1.48 (1.10 - 1.99) | 0.009 | 1.75 (1.25 - 2.46) | 0.001 |

Model 1: Adjusted for age, sex, BMI, renal function, blood pressure, diabetes, smoking status, and non-HDL cholesterol.
Model 2: Adjusted for age, sex, BMI, renal function, blood pressure, diabetes, smoking status, non-HDL cholesterol, and a history of vascular disease.
